# Effectiveness of endovascular thrombolysis in acute mesenteric vein thrombosis

**DOI:** 10.1186/1471-2318-11-S1-A35

**Published:** 2011-08-24

**Authors:** M Milone, G Di Minno, MND Di Minno, V Iaccarino, P Venetucci, F Milone

**Affiliations:** 1 Department of Surgery, Orthopedics and Emergency, Unit of General Surgery, “Federico II” University, Naples, Italy; 2 Department of Clinical and Experimental Medicine, Reference Centre for Coagulation Disorders, “Federico II” University, Naples, Italy; 3 Department of Radiology and Radiotherapy, Unit of Cardiovascular and Interventional Radiology, “Federico II” University, Naples, Italy

## Background

Mesenteric vein thrombosis (MVT) is a rare, often lethal, entity that accounts for approximately 10-15% of all cases of mesenteric ischemia [1,2]. Current indications for surgery in patients with acute MVT include signs of peritonitis, bowel infarction and hemodynamic instability.

In all other cases, long-lasting anticoagulation is the strategy of choice [3,4], patients with MVT have a fairly good prognosis and long-term outcomes once appropriate anticoagulation is achieved [4,5]. At variance with the slow onset of recanalization that takes place during anticoagulation, thrombolysis leads to a rapid re-opening of a vessel, with immediate tissue reperfusion [4].

## Materials and methods

We have followed up each for at least 3 years. 32 MVT patients (Table 1), 18 of whom (treated group) had undergone percutaneous transhepatic thrombolysis and mechanical thrombectomy prior to starting long-lasting warfarin treatment. The other 14 patients (control group) received only warfarin treatment. In each case and for each patient, the rate of surgical approach (bowel resection) and the rate of long-term mesenteric-portal hypertension was evaluated.

**Table 1:** Clinical diagnosis on admission

	Control Group	Treated group	Statistical significance
**Asa** Asa 2 Asa 3	4 10	7 11	P = 0.712

**Thrombosis localization** Mesenteric Mesenterico-portal Spleno-mesenter-portal	5 7 2	7 8 3	P = 0.950

**Duration of symptom** **≤** 2 day ≤ 7 day ≤ 14 day	1 9 4	2 10 6	P = 0.865

## Results

**Figure 1:**
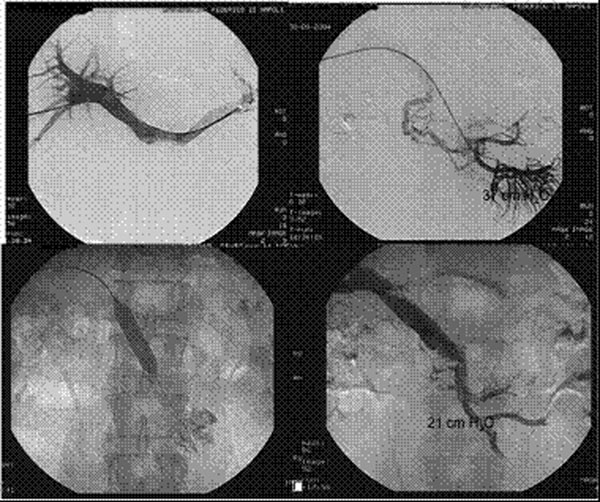
Percutaneous Thrombolysis and venoplasty

**Figure 2:**
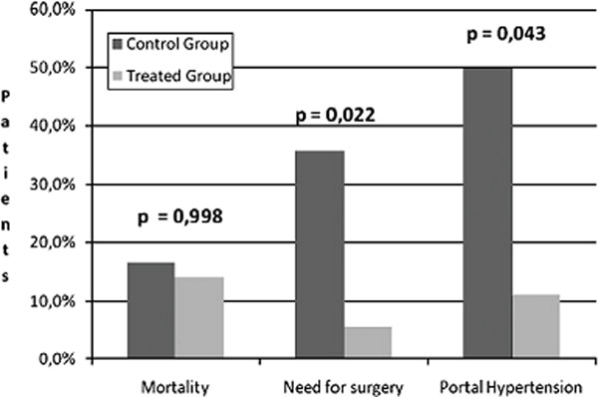
Results of the trial following the pharmacological treatment

In 16/18 patients (88.8%) following the percutaneous treatment, flow restoration in the thrombosed mesenteric vein was documented by direct portal venography (Fig. 1). All patients with successfully recanalized MVT did not develop recurrent episodes during the long-lasting (1 year) oral anticoagulation therapy. The 30-day mortality rate was similar in the two groups (p=0.998). Bowel resection was needed in 1 patient (5.5%) in the treated group and in 5 patients (35.7%) in the control group (p=0.022 as to the rate of short-term surgical sequelae). A significant difference was also found as to long-term sequelae, especially portal hypertension (7/14 patients in the control group, 50.0%; 2/18 patients in the treated group, 11.1%; p=0.043) (Fig. 2).

## Conclusions

MVT is still a serious disease, with a high mortality rate (25-35%), mostly related to transmural necrosis and bowel perforation due to the delay in diagnosis [1,6]. In the absence of major clinical signs and symptoms, the severity of bowel ischemia on admission is based on the evaluation of bowel wall thickness by contrast-enhanced CT scan (90% sensitivity). Macroscopically infarcted small bowel without transmural necrosis is potentially reversible with long-lasting anticoagulation [1,7-10]. Encouraging results of endovascular thrombolytic treatments have been reported in literature [11,12]. According to our results, when administered promptly, endovascular intervention using percutaneous transhepatic thrombolysis and mechanical thrombectomy appears to have a lower rate of early and late complications compared to warfarin treatment alone.
